# The Effect of Safety Leadership on Safety Participation of Employee: A Meta-Analysis

**DOI:** 10.3389/fpsyg.2022.827694

**Published:** 2022-06-16

**Authors:** Linyi Zhao, Daojian Yang, Suxia Liu, Edmund Nana Kwame Nkrumah

**Affiliations:** School of Management, Jiangsu University, Zhenjiang, China

**Keywords:** safety leadership, safety participation, safety behavior, safety climate, meta-analysis

## Abstract

Recently, the promotion of safety participation (SP) has become a hot spot in behavioral safety research and safety management practice. To explore the relationship between safety leadership (SL) and SP, a theoretical model was established and 33 articles (35 independent samples) on work safety from 2000 to 2021 were selected for a meta-analysis. By evaluating the impact of SL, which incorporates transformational, transactional, and passive leadership styles, on work safety. The results show that SL has a positive impact on both safety climate (SC) and SP. Both safety transactional leadership (STAL) and safety transformational leadership (STFL) positively impact SP, and the impact of STFL is greater, while safety passive leadership (SPL) has no impact on SP. The study establishes that SC plays a partial mediating role between transformational SL and employee SP. Under the condition of a developed economic level or high-risk industry, SL indicated a greater influence on SP. Hence, it is recommended that when enhancing the SP of employees, the influence of the macro environment and SC should not be undermined.

## Introduction

Work safety has always been the hot spot in academic research and safety management practice (Gao et al., 2019). Several studies in this field have focused on employees’ safety compliance. In as much as safety compliance has a positive impact on an enterprise’s work safety, it is not enough to improve the work safety level. By examining data analysis on employee safety behavior and enterprise safety performance, it was found that safety performance still does not reach the ideal level even when enterprises took measures to improve safety compliance (Clarke, 2006; Mullen et al., 2017). Thus, to further improve the level of work safety, the concept of employee safety participation (SP) remains inseparable. Compared to safety compliance, SP is usually considered as employees voluntarily participating in safety-related activities, such as attending safety meetings, taking the initiative to put forward safety improvement suggestions, helping co-workers to stay away from risks, and so on. In this regard, many studies have recognized the significant role of SP (Gunningham, 2008; Liu et al., 2020; Yang et al., 2021). Notably, SP can not only facilitate the implementation of enterprise safety management but also can reduce safety accidents (Wu et al., 2008). Considering the unique characteristics of grassroots employees, who are most directly related to production activities, employees themselves are likely to be more closely related to the involvement in safety-related activities, hence the positive effect of employee SP on enterprises may be greater.

Through the development of economies and the current interest in practicing safety management, a few safety leaders have realized the importance of SP, with some in full swing to take top-down actions that can improve and better safety performance as a whole. Temporarily, the theoretical value of SP research has gradually been revealed, as research that attempt to explore the influencing factors of SP has increased. Most safety-related studies choose to study the antecedents of SP from the internal factors of the organization, and one of the variables which stood out is safety leadership (SL). This is because the safety leader in the top decision-maker of the enterprise’s safety production management. The behavior of the safety leader is directly related to the organization’s safety production, which can guide the behavior of employees and have a great impact on the enterprise’s safety environment (Day and Miscenko, 2016; Jiang et al., 2017). Deluga (1992) pointed out that leaders’ behaviors such as humanistic management and efficient work, can promote the formation of a high-quality relationship between leaders and employees, so as to stimulate employees’ interest in work and achieve higher goals. As safety leaders are the main superiors of grass-roots employees, their tremendous role in accident prevention remains very critical. Safety leaders can as well influence employees’ behavior through value shaping, vision incentive, humanistic care, and innovation drive (Avolio et al., 2009).

In as much as the concept of SL has widely been preached. With the deepening of theoretical research, more variables were involved, including safety climate (SC), safety motivation, and so on. However, with the increase in the number of relevant studies, the differences are becoming larger and larger, and different researchers have drawn different conclusions. Considering the differences in the relationship between SP and SL researchers have attempted to carve different understandings of SL and SP, hence, dividing the concept into several dimensions. For instance, a study by Smith et al. (2016) showed that safety passive leadership (SPL) has a negative influence on SP, while on the contrary, Xue et al. (2020) thought the direction should be positive. Be as it may be, the research samples are based on different countries, different industries, and different times, and may account for these discrepancies. In a study on the construction industry in the United Kingdom (Conchie and Donald, 2009; *r* = 0.38), the correlation coefficient between SL and SP is much lower than that in China (Wu et al., 2008; *r* = 0.81). Another evidence is the correlation between SL and SP in manufacturing (Oah et al., 2018) is triple that in the chemical industry (Zohar and Luria, 2010). There may be several reasons for the discrepancy, one of which comes from the research background, such as different employee behavior patterns in different economic backgrounds and different industries.

These different conclusions may easily confuse policymakers and researchers (or readers). With the development of safety behavior research and scientific progress, it is important to clarify the differences established in previous research. In this study, the adoption of the meta-analysis method can easily address these discrepancies. As a mathematical tool, the use of the meta-analysis can integrate many single research data, so as to find general conclusions and differences. To complete this integration process, the concepts of SL and SP were clearly defined by reviewing relevant theories and previous research. Second, a new conceptual model was established to further explore the relationship between SL and SP, and find mediators or moderators and the roles that may exist between them. The study therefore presents a review of 33 research findings (35 independent samples) on the relationship between SL and SP within the period 2000 and 2021 and explains the influence of some potential mediating and moderating effects on them. Finally, the research results are sorted out and the conclusions are drawn, practical and effective suggestions to improve employee’s SP are as well provided.

## Theory and Hypotheses

### Safety Leadership

Safety leadership originates from general leadership theory. Some researchers think that SL is a process of influencing all employees to chase the safety goal of their organization (e.g., Avolio et al., 2009; Wu et al., 2011). Tao et al. (2020) reviewed the theoretical research of domestic and foreign scholars on SL from 1999 to 2019, and sorted out its evolution process. And Wu et al. (2008) also emphasized that safety leaders should have enough leadership skills on safety (e.g., safety knowledge and decision-making ability, etc.), and utilize these skills to improve the whole safety environment. Based on these previous studies, this present study defines SL as an influence process in which the safety leader improves the work safety environment of the enterprise, guides, or requires employees to regulate their own safety behaviors, and helps them obtain the support of the organization to achieve the overall safety goal of the enterprise.

In Omnibearing Leadership Theory, Bass (1985) divided leadership into three different dimensions, namely, transformational leadership, transactional leadership, and passive leadership. By adopting the concept of this theory, this study divides SL into three dimensions, which incorporate safety transformational leadership (STFL), safety transactional leadership (STAL), and SPL. STFL describes a relatively ideal state in which the leader instills confidence and values in followers, motivates others, and describes the vision so that followers recognize and take actions that are consistent with the organization’s goals (Barling et al., 1996). STAL refers to the leader monitoring the safety behavior of employees, caring for employees individually, discussing safety issues with employees, and actively managing safety before an accident occurs. SPL includes management-by-exception leadership and laissez-faire leadership (Avolio et al., 1999), and it is related to an event were safety leaders generally do not take the initiative to participate in safety management but rather prefer to take action after the occurrence of serious safety problems or accidents, and severe punishment for those who made mistakes.

### Safety Climate

The concept of SC was first proposed by Zohar (1980) to reflect the perception of the organization’s safety environment by the members of the organization. Based on the theory of organizational behavior, its content is gradually enriched (Guldenmund, 2000). The important role of SC had been widely recognized, and it became a variable that can represent a unique safety feature of an organization and not be ignored to measure the safety situation of enterprises (Mullen, 2004). Broadly speaking, a SC can represent all the factors within an enterprise that are related to the safety environment, which includes both the “hard” and “soft” part (Kalteh et al., 2021). But this general description is not conducive to scientific research, so researchers prefer to reinterpret the SC based on their understanding. In other interpretations, the SC is regarded as a variable almost equivalent to the safety culture, and it is defined as a current reflection of safety culture (Mearns et al., 2003). However, a SC focuses more on environment and perception, and some factors that do not form a fixed culture can also be considered in the SC (Casey et al., 2017). Though there are indeed a lot of overlaps between these two variables, and some researchers continue to use safety culture to represent SC, this study still holds the view that they are two independent variables. Thus, based on the consensus of previous studies, safety communication between the safety leader and the employees, the safety concern of the safety leader to the employees, and the dissemination of safety concepts can all be regarded as the standard to measure the safety climate (Alruqi et al., 2018). This study, therefore, defines the SC as the common perception of internal personnel on organizational safety features, which is also part of the consensus of most researchers.

### Safety Participation

The concept of SP was first proposed by Griffin and Neal (2000), which refers to the behavior of employees voluntarily participating in safety-related activities and attending safety meetings. They further enriched the scope of SP, except the voluntary participation behavior activities, which include helping co-workers solve the problem of work safety and taking advice from superiors to help improve the safety environment level in companies (Neal and Griffin, 2006). However, with the increasing number of researchers in this subject area, some variables with similar content such as safety citizenship behavior, safety extra-role behavior, and so on appeared. Safety citizenship behavior emphasizes the result of this behavior is beneficial to the organization, but the safety extra-role behavior emphasizes that this behavior is non-post-responsibility, and employees have the willingness to take the initiative. Although the definitions of these variables are slightly different, they have roughly the same meaning, which is they all emphasize that in addition to obeying the requirements of the enterprise and following the safety rules and regulations, employees voluntarily and actively make safety behaviors conducive to the safety performance of the organization. To make the definition of SP clearer, Curcuruto et al. (2015) have made a more detailed division of it. SP can be divided into safety pro-social behavior and safety initiative behavior according to the object of an action. Pro-social safety behavior refers to the social behavior among grass-roots employees, including help, advice, and protection. Safety initiative behavior refers to the spontaneous behavior of employees, including those who actively participate in safety training to improve the safety level of the working environment (Curcuruto et al., 2015). In other words, SP describes a behavior that does not directly improve workers’ personal safety behavior, but what indirectly makes contributions to the change of the safety environment of the enterprise (Martínez-Córcoles et al., 2012). Like Martínez-Córcoles (2012), when defining SP, quite a few researchers also choose to describe characteristics and functions.

Based on the definition of previous researchers (Neal and Griffin, 2006; Curcuruto et al., 2015; Curcuruto and Griffin, 2018), this study defines SP as the behavior of grass-roots employees to voluntarily participate in the work safety of an enterprise, such as participating in safety activities, attend safety meetings, taking the initiative to put forward safety improvement suggestions, and helping co-workers to stay away from risks among other safety-related behaviors.

### The Influence of Safety Leadership on Safety Climate

Earlier studies on the relationship between general leadership and organizational climate speculated the relationship between SL and SC, and then proved the existence of such relationship through empirical research. Krause (2004) believed SL can improve the safety awareness of employees and strengthen the SC of the organization. Kapp (2012) asserted that SL significantly affects SC and indicated that the leadership practice of daily interaction and guidance with employees can effectively improve SC.

Subsequent studies gradually revealed the relationship between different leadership styles and SC. According to leader’s safety behavior, Du and Sun (2012) divided SL into two dimensions. Then, by proving every leader’s safety behavior is related to the SC, he draws that two styles of SL are both related to the SC. Conchie (2013) found that transformational SL can improve employees’ perception of the SC. In other papers (e.g., Den Hartog et al., 1997; Clarke, 2013; Shi, 2021), researchers made comparative studies on the relationships between STFL and STAL with other variables and concluded that transformational leadership has a more significant impact on SC. On the contrary, because SPL is generally considered as a less effective style of leadership behavior (Berry et al., 2007), there are few relevant studies related to it. Bass’s leadership theory, however, argued that SPL should bring passive influence to the organization. But in the study of Kelloway et al. (2006), he put out an unexpected conclusion, indicating that passive leadership contributes incrementally to the prediction of safety-related variables. Nonetheless, the conclusion of the majority of articles maintained the original judgment. Jiang and Probst (2016) found that STFL strengthened SP whereas SPL weakened it. In a recent study from China, safety-specific passive-avoidant leadership negatively affects the safety compliance behavior, which is not conducive to the safety environment of enterprises (Liu et al., 2021). Therefore, we similarly expected that:

H1: Safety leadership is positively related to safety climate.
H1a: Safety transformational leadership is positively related to safety climate.
H1b: Safety transactional leadership is positively related to safety climate.
H1c: Safety passive leadership is negatively related to safety climate.

### The Influence of Safety Leadership on Safety Participation

Leadership has been identified as an important factor that influences SP. SL can enable employees to participate in the work of the enterprise more actively and efficiently so as to make them responsible for the work safety of the enterprise (O’Dea and Flin, 2001). Hofmann et al. (2003) found that in the army when leaders show concern for their employees, employees are more likely to do safety extra-role behaviors to show positive feedback to their leaders. In the manufacturing industry, Clarke and Ward (2006) found that the effective implementation of safety goals by leaders has a significant direct positive impact on SP. Similarly, the same results have been found in eastern countries (e.g., Bian et al., 2019; He et al., 2021). That is to say, under different national and industrial backgrounds, SL is related to SP. Therefore, we put forward the hypothesis that SL is positively related to SP (H2).

Like the impact of SL on the SC, different leadership styles and leader behaviors both have different influences on the SP of employees. Both transformational leadership and transactional leadership can play a significant role in SP, but the effect of transformational leadership is more obvious (Clarke and Ward, 2006; Mullen et al., 2017). In addition, when leaders strictly abide by and implement the safety management system, they can improve employees’ sense of belonging to the organization and promote the formation of internal consensus (Wu et al., 2008). However, Zohar (2002) firstly found that the impact of SPL on safety behavior is opposite to transformational leadership and transactional leadership. In addition, Mullen et al. (2011) found that passive leadership has a negative impact on SP. Thus, we predicted that:

H2: Safety leadership is positively related to safety participation.
H2a: Safety transformational leadership is positively related to safety participation.
H2b: Safety transactional leadership is positively related to safety participation.
H2c: Safety passive leadership is negatively related to safety participation.

### The Mediating Role of Safety Climate Between Safety Leadership and Safety Participation

A positive SC can promote the employees’ safety behavior, reduce risks, and improve safety practices (Felknor et al., 2000), and it can also promote employees to actively discuss safety issues and consequently improve employees’ SP significantly (Hon et al., 2014). The mediating role of SC in the relationship between leaders’ behavior and employees’ behavior has also been supported in many empirical studies (Barling et al., 2002; Clarke and Ward, 2006; Kelloway et al., 2006). In the subsequent empirical research on SP, similar conclusions were obtained. Lu and Yang (2010) believed that enterprise safety leaders have an indirect influence on safety behavior through the mediating role of the work atmosphere. Lee et al. (2019) found that SL, SC, and SP were related to each other, and SC played a mediating role in them. Abdullah et al. (2021) showed that SL affects SP through the mediating role of SC and safety motivation. Therefore, SC can not only directly affect SP but also play a mediating role between SL and SP, hence hypotheses 3 and 4 are proposed.

H3: Safety climate is positively related to safety participation.
H4: Safety climate mediates the relationship between safety leadership and safety participation.

### The Moderating Role of Economic Level and the Industry Risk Degree

According to the theory of leader-member exchange (LMX), the exchange relationship between leaders and members is different in the different social backgrounds (Dulebohn et al., 2012). Moreover, under the infiltration of the organizational environment, people’s internal and external environment will be integrated, and the integration result will interfere with employees’ behavior (Zhang et al., 2010). To reduce the research error, two moderate factors (economic level and industry risk degree) were selected to explore whether there is a moderating effect of SL on SP. Different economic levels may lead to different impacts of SL on employees’ SP (Nahrgang et al., 2007). At a high economic level, the maturity of enterprises and the education level of employees are higher. Generally speaking, enterprises in developed countries pay more attention to work safety and have better safety management. Chen and Li (2019) proposed that people’s behavior types correspond to cognitive types, which explains that employees with good knowledge levels and learning abilities tend to be well-behaved. However, for some enterprises in developing countries not only is the safety management poor but also the employees’ safety awareness is low. Therefore, in places with lower economic development levels, employees have more habitual violations and it is more difficult to improve employees’ SP. On the other hand, the industry risk level will also have an impact on the relationship between SL and SP. Special operators in high-risk enterprises generally have additional safety training, and if they operate incorrectly, serious consequences may be caused. So employees in high-risk industries are more cautious and have better safety performance (Lingard, 2002). On the contrary, people in low-risk industries may ignore SP because the improvement of SP has a less obvious effect on improving safety performance. Hypotheses 5 and 6 are proposed as,

H5: Economic level plays a moderating role in the relationship between SL and safety participation.
H6: Industry risk degree plays a moderating role in the relationship between safety leadership and safety participation.

**FIGURE 1:**
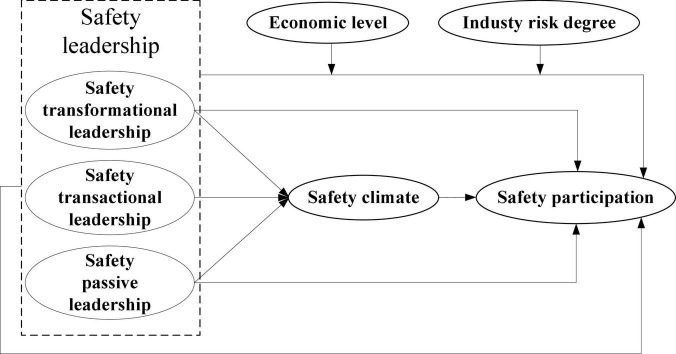
Conceptual model.

The final conceptual model is shown in Figure 1.

## Methods and Samples

### Literature Search

To ensure that the samples are not missed, three rounds of selection and two methods are used. The first round of selection was done manually. After reading several papers, a general topic scope and a clear selection standard were determined.

Next, a computerized search in Web of Science, Science Direct, and CNKI was used to infiltrate published articles, which include SL (styles), transformational leadership, transactional leadership, passive leadership SC, SP, safety citizenship behavio(u)r or safety behavio(u)r between 2000 and 2021. In addition, for preventing missing any possible samples, we did a manual re-search of main researchers and major journals such as safety science, accident analysis, and prevention in work safety.

After these articles are collected, the third round of selection begins. Studies identified must satisfy 4 demands at the same time. First, all of them must include at least two of the three aspects among SL, SC, and SP; second, all data must be measured at the individual level and drawn from occupational samples; third, each study that was searched from databases must record publish date, effect sizes on variables of interest (correlation coefficients, *t*-value or *p*-value), sample sizes, and other reliable information; last, for the literatures repeatedly published, the data shall be subject to the literature containing more variables.

In the end, 35 independent samples (*N* = 15749) from 33 articles were selected for this study, and the detailed information is shown in an Supplementary Appendix at the end of this paper. The sampling distribution of the effect size of each study followed the normal distribution with known sampling variance, which is in line with the basic conditions of meta-analysis.

### Coding for Studies

Considering that the expressions of SL, SC, SP are different in various studies, the search scope of relevant keywords was expanded in this study to ensure the accurate identification of variables. The main codes are shown in Table 1.

**TABLE 1:** Coding for studies.

Variable	Dimension	Main code
Safety leadership	Safety transformational leadership	Safety-specific transformational leadership
		Safety inspiration
		Rational persuasion
		Safety vision empowering leadership
	Safety transactional leadership	Safety-specific transactional leadership
		Safety monitoring and control
		Personal safety concerns and consultations
		Management by exception active
	Safety passive leadership	Safety passive leadership management by exception passive
		Laissez-faire
Safety climate		Safety culture
		Perceived safety climate Safety attitude of managers Management commitment to safety Managerial safety values
Safety participation		Worker’s cooperation on safety
		Feedback and advice on safety
		Safety citizenship behavior Participate in safety activities Proactive safety behavior

In the coding process, there are still many ambiguous words. According to the definition of variables in this article and the suggestions of experts, they are encoded as variables with the closest meaning or deleted, which are not listed here because of their low frequency.

Then, each sample is used as a data unit. The author, year, sample size, and effect value were encoded into comprehensive meta analysis (CMA). If the correlation coefficient between the dimensions of the variable is reported in the study, the final effect value is calculated using CMA software.

## Results

### Test for Publication Bias

**FIGURE 2:**
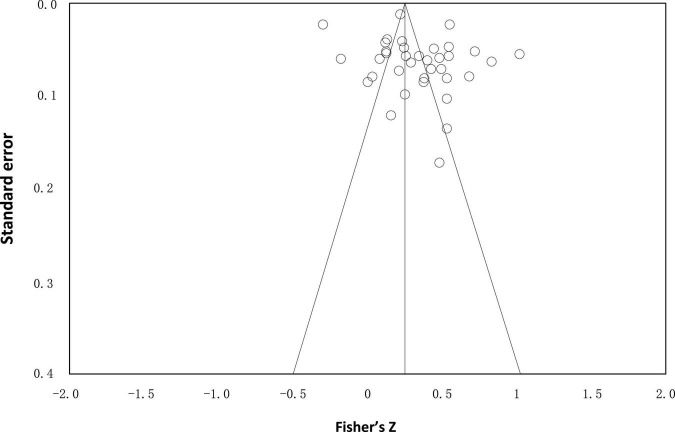
Funnel plot of standard error by Fisher’s *Z*.

For ensuring the reliability of statistical reanalysis, a publication bias test that included all samples is needed before meta-analysis. Funnel plot and classic fail-safe N are used to test publication bias in this study and the results are displayed in Figure 2 and Table 2. In Figure 2, all samples’ standard error by Fisher’s *Z* is formed into a funnel plot, and in Table 2, values that have been calculated by the classic fail-safe *N* method are listed.

**TABLE 2:** The analysis of classic fail-safe *N*.

Items	Value
*Z*-value for observed studies	36.473
*P*-value for observed studies	0.000
Alpha	0.050
Tails	2.000
Z for alpha	1.960
Number of observed studies	35.000
Number of missing studies that would bring *p*-value to >alpha	2086.000

In Figure 2, except for a few samples, most of the points are concentrated at the top of the funnel plot, which means the sample in this paper is basically not biased. At the same time, most of them are evenly distributed on the left and right sides of the middle line, which means the observed overall effect is robust. Therefore, the funnel plot shows that the selected samples do not have publication bias and meet the conditions for further study.

In the classic fail-safe *N* method, although there is no way to intuitively see whether there is deviation, it can be determined according to the size of the data.

After comparison and identification, the data in Table 2 reprove this study has no publication bias. In the classic fail-safe *N*-test, the bigger the number of missing studies, the more reliable the conclusion of the meta-analysis is. When *p* = 0.000 and α = 0.05, the number of missing studies that would bring a *p*-value bigger than alpha is 2086. In other words, to reverse the conclusion of this study, at least 2086 opposite or useless related studies are needed. During the literature search, it is impossible to find such a large number of samples, there was no publication bias in this paper. Thus, the scientific nature of the research is guaranteed and this research can be continued.

### Test of Heterogeneity

The purpose of the heterogeneity test is to examine the degree of difference between independent studies and to calculate whether it is mergeable. If there has no heterogeneity among these samples, the fixed-effect model should be selected. On the contrary, the random effect model should be selected to optimize the overall effect.

*Q*-value and *I*^2^ are the common heterogeneity indicators in meta-analysis. When the *Q*-value is bigger than the critical value and *I*^2^ is bigger than 75%, the study is considered to be heterogeneous. Conversely, there was no heterogeneity in this study. The calculated data is recorded in Table 3.

**TABLE 3:** Fixed effects and random effects meta-analysis.

Model	Effect size and 95% interval				Z	Heterogeneity				Tau-squared			
	NS	PE	L	U		*Q*-value	df (Q)	*P*	*I* ^2^	Tau^2^	SE	Variance	Tau
F	35	0.26	0.25	0.28	33.70	1971.50	34	0.00	98.28	0.14	0.06	0.00	0.37
R	35	0.36	0.25	0.47	5.95								

*NS, number studies; PE, point estimate; L, lower limit; U, upper limit; SE, standard error.*

According to Table 3, when the *p*-value equals zero, both *Q*-value and *I*^2^ are bigger than the standard values (*Q*-value = 1971.50 > 34, *I*^2^ = 98.28 > 75%), which means heterogeneity exists. Therefore, the random-effects model should be chosen.

### Overall Effect Size Based on Random Effect

Test results of hypotheses between SL, SC, and SP are shown in Tables 4, 5. Table 4 test the overall relationships and Table 5 shows more details on the sub-variables of SL. It is worth mentioning that, although several independent samples are contained in one article, it is necessary for one article to only have one index when doing summary analysis. That is to say, several independent samples from the same paper are firstly integrated to calculate a comprehensive correlation coefficient, and then analyzed together with other samples. Basically, each hypothesis takes the same data processing method.

**TABLE 4:** Overall effects of the relationship among SL, SC, and SP.

Relationship	NS	PE	95% CI		*Z*-value	*P*-value	Heterogeneity				SE
			L	U			*Q*-value	Df (Q)	*P*-value	*I*-squared (%)	
SL-SC	18	0.307	0.138	0.459	3.477	0.001	1243.522	17.000	0.000	98.633	0.087
SL-SP	21	0.359	0.246	0.461	5.939	0.000	483.718	20.000	0.000	95.865	0.033
SC-SP	14	0.535	0.442	0.617	9.592	0.000	196.412	13.000	0.000	93.381	0.026

*NS, number studies; PE, point estimate; 95% CI, confidence interval around effect size, L, lower limit; U, upper limit; SE, standard error.*

**TABLE 5:** Test results of model path coefficient.

Relationships	NS	TN	PE	95% CI		*Z*-value	*P*-value	Heterogeneity				SE
				L	U			*Q*-value	Df (Q)	*P*-value	I-squared (%)	
STFL-SC	15	8072	0.531	0.386	0.650	6.296	0.001	741.464	14.000	0.000	98.112	0.078
STAL-SC	7	1641	0.493	0.133	0.738	2.604	0.009	391.6443	6.000	0.000	98.468	0.197
SPL-SC	10	4453	−0.244	−0.450	−0.015	−2.084	0.007	437.5093	9.000	0.000	97.943	0.094
STFL-SP	17	5195	0.456	0.387	0.521	11.420	0.000	136.565	16.000	0.000	98.284	0.013
STAL-SP	13	4574	0.347	0.139	0.527	3.189	0.001	647.213	12.000	0.000	88.146	0.084
SPL-SP	8	2400	0.126	−0.044	0.289	1.458	0.145	120.711	7.000	0.000	94.201	0.034

*NS, number studies; TN, the total number involved; PE, point estimate; 95% CI, confidence interval around effect size; L, lower limit; U, upper limit; SE, standard error.*

In Table 4, all relationships were in the direction hypothesized, and the differences were not significant at the 5% level, therefore, hypotheses 1, 2, and 3 can be considered fully supported. According to the results, SL positively affected SC (H1) (PE = 0.307, *p* < 0.01) and SP (H2) (PE = 0.359, *p* < 0.01); SC is more positively affected SP than SL (H3) (PE = 0.535 > 0.359, *p* < 0.01). This may be because people are more susceptible to the influence of environmental changes. It enlightens leaders not to ignore the importance of creating a better SC when promoting employees’ SP. Meanwhile, the hypotheses of H1a, H1b, and H1c are all supported. Both STFL (H1a) (PE = 0.531, *p* < 0.01) and STAL (H1b) (PE = 0.493, *p* < 0.01) had a positive effect on SC, while SPL (H1c) (PE = –0.244, *p* < 0.01) had a negative effect on SC. The same situation applies to other hypotheses: a significant, but smaller, effect size was found in STFL-SP (H2a) (PE = 0.456, *p* < 0.01) and STAL-SP (H2b) (PE = 0.347, *p* < 0.01). However, in the test of H2c, the data were not statistically significant (PE = 0.126, *p* = 0.145 > 0.01). This may be because small sample sizes or SPL is inclined to post-management and does not focus on the behavior process of SP.

Comparing these results, STFL has a stronger impact on SC and SP than STAL, which indicates that STFL may be a more effective leadership style within the company.

### The Mediating Effect of Safety Climate

The effect values of path SL-SC-SP and path SL-SP are compared to verify whether SC has a mediating role. After manually filtering, nine articles containing SL, SC, and SP were selected. In the range of error allowable, if the effect of path SL-SC-SP is more obvious than that of SL-SP, SC is considered to have a mediating effect. As can be seen from the data in Table 6, *p*-value is statistically significant in both random effect models (*p* = 0.000 < 0.01), and the value of the PE effect is bigger in path SL-SC-SP (0.357 > 0.331), which means SC plays an intermediary role in the relationship between SL and SP. The mediating effect of SC exists, and so H4 is supported.

**TABLE 6:** The mediating effect of safety climate.

Path	TN	NS	PE	95% CI		*Z*-value	Heterogeneity				SE
				L	U		*Q*-value	Df (Q)	*P*-value	I-squared (%)	
A: SL-SC-SP	6492	9	0.357	0.143	0.539	3.189	224.644	8	0.000	96.439	0.067
B: SL-SP			0.331	0.157	0.485	3.634	235.755		0.000	95.758	0.047

*NS, number studies; PE, point estimate; 95% CI, confidence interval around effect size; L, lower limit; U, upper limit; SE, standard error.*

The role of a good SC between employees and leaders is like glue, which can increase the communication of safety information and reduce potential conflicts. SC should be highly valued in daily safety management. Leaders can create an environment that encourages employees to report safety issues and deal with them in time, so a positive SC can be formed gradually.

### Moderator Analysis

In the conceptual model, two moderators are introduced in the study of the relationship between SL and SP: economic level and industry risk degree. Based on different types of work, samples are divided into high-risk industry (such as coal mines, chemistry, oil, and construction) and low-risk industry (such as army, service, trade a mixture of industries, and unknown industry) groups. Then, following the international standard, these samples were reclassified into developed countries (or regions) and developing countries (or regions). Meta subgroup analysis was performed, and the results are shown in Table 7.

**TABLE 7:** Results of moderators with subgroup analysis.

Variable	Category	NS	TN	PE	95% CI		Test of null (2-Tail)		Heterogeneity				SE
					L	U	Z	*P*	*Q*-value	Df (Q)	*P*	*I*^2^ (%)	
Economic level	Developed	24	12471	0.218	0.201	0.234	24.616	0.00	1633.999	23	0.00	98.592	0.084
	Developing	11	3278	0.425	0.397	0.453	25.882	0.00	197.529	10	0.00	94.937	0.032
Industry risk degree	High-risk	22	7055	0.465	0.447	0.483	42.133	0.00	568.289	21	0.00	96.305	0.035
	Low-risk	13	8694	0.080	0.059	0.101	7.449	0.00	708.361	12	0.00	98.306	0.084

*NS, number studies; TN, the total number involved; PE, point estimate; 95% CI, confidence interval around effect size; L, lower limit; U, upper limit; SE, standard error.*

Indeed, both two variables have moderating effects, so H5 and H6 are supported. The influence of SL in developing economies (PE = 0.425, *p* < 0.01) on SP is greater than that in the developed economy (PE = 0.218, *p* < 0.01), and the influence of SL in high-risk industry (PE = 0.465, *p* < 0.01) on SP is obviously greater than that in low-risk industry (PE = 0.080, *p* < 0.01).

Since most enterprises under the developing economic level are pyramid-shaped organizations, the whole environment is more traditional, so safety leaders have a greater influence on employee behavior than under the developed economic level. Moreover, in high-risk industry, the dangers of work makes employees pay more attention to work safety, and they are more likely to make behavioral changes under SL.

## Discussion

### Discussion of Results

When people just study the influencing factors of SP, they often continue the previous research idea of safety behavior, that is, they focus on the external influencing factors, especially the influencing factors within the organization. These studies are basically similar in terms of the theoretical basis and research process, but their conclusions are dissimilar. So this study used meta-analysis to examine the differential effects of SL (including STFL, STAL, and SPL), and SC on SPL, including the effect of SC as a mediator. Furthermore, the moderating effect of economic level and industry risk degree were examined.

#### Discussion of Results on the Influence of Safety Leadership on Safety Climate and Safety Participation

Like most previous studies, the impact of SL on SC is positive and significant, and different dimensions under it show different influences on SP. Except for SPL, which showed significant negative effects, the other two SL styles showed positive effects. In addition, compared with the STAL, the results showed that the STFL has stronger promotion and smaller statistical point estimate bias on the SC. This provides an idea for improving the overall safety environment of enterprises.

Safety leadership was found to have a valid and generalizable relationship with SP, as well as its three dimensions (STFL, STAL, and SPL). As a whole, SL leads to higher levels of SP, and both STFL and STAL have made contributions in this relationship. In particular, the more transformational the leadership is, the greater the improvement of SP will be. Unfortunately, the hypothesis about SPL inhibits SP is not supported. As mentioned above, it is possible that this is a matter of sample selection, but it is more likely that different people react differently to post safety management. Some employees choose to take active safety activities on workdays because they are afraid of the severe management and punishment of their leaders after safety accidents, while other employees do not care about SP because of the loose management of the leaders on workdays. This also provides a suggestion for us to study the influence between SPL and SP in the future. The influencing factors of SP should be considered comprehensively according to the different characteristics of research samples.

#### Discussion of Results on the Mediating and Moderating Effect

The results prove the mediating role of SC between the relationship of SL and SP. SL can indeed improve the overall environment of the organization. As for the SC, which is an aspect of the organizational environment, it will have an imperceptible impact on the employees in this environment, that’s why the SC–SP relationship has the same direction as SL–SC. Kalteh et al. (2021) points out that SC plays a crucial role in enterprises, and there is an interaction between SC and safety behavior. And whether leaders or employees, one of the results of behavior improvement is that the overall safety environment and safety performance of enterprises have been improved to a higher level (Kalteh et al., 2021). The mechanism of this interaction still needs to be further verified, but it suggests the possibility that the safety atmosphere plays more roles than mediating role.

Moderator analysis indicates that both industry risk degree and economic level have a significant effect on the overall effect, and the moderating effect of economic level is more significant. Therefore, when safety leaders improve SP, they should think more comprehensively, because SL and improvement of the social-economic environment are both important.

### Limitations

Although following the scientifically and prudent studying steps and striving for perfection, limitations still inevitably exist. First, samples may be missing. As the whole study takes a long time, the gap between the end time of sample screening and the writing time is nearly half a year, so the latest sample may not be included. In addition, in the second round of selection, only mainstream English databases were used, and most of the selected samples were high-level articles, excluding ordinary journals in non-English speaking countries. Second, compared with the number of studies on other kinds of safety behaviors, the number of SP is small. And many data in the meta-analysis have a cross-sectional nature, which means that although several hypotheses are supported in this paper, the direction of the results shown in the model may not be the real direction of the relationship between variables (Clarke, 2006). To adjust it requires further longitudinal testing. However, from the fact that the results of this paper are consistent with most studies, there may be no deviation. Third, individual behavior is the result of the interaction between internal personal factors and external environmental factors (Bandura, 1986; Locke and Bandura, 1987). Therefore, the actual model of SL–SC–SP may be much more complex, and it does not eliminate the possibility that some potential moderators may be ignored. It can be boldly assumed that national culture and personal characteristics will have a certain impact on employees’ SP. Similarly, social and cultural background and leadership characteristics will also affect SL. With the deepening of research, researchers should consider more comprehensively and gradually optimize the SL–SP conceptual model to make it more realistic.

### Implications

This article makes certain contributions. First, this study combs previous studies and makes the definition of SL and SP clearer. For example, it describes the theoretical development history of SL and explains why it is defined as three dimensions (STFL, STAL, and SPL). The similarities and differences between SP behavior and other variables (such as safety citizenship behavior and safety extra-role behavior) are also pointed out. The emerging new definitions and dimensions are a generalization and re-understanding of the consensus of most studies, which is helpful for researchers to understand relevant concepts quickly.

Second, the conceptual model of SL–SP was formed, and the inconsistent conclusions in previous studies were clarified through meta-analysis. Such as STFL does have a more significant impact on employees’ SP than safety transactional leadership and the relationship between SPL and SP was not supported. To a certain extent, these findings may be helpful for enriching the theoretical research in the field of work safety and providing a theoretical guidance for future research.

Third, this article can also contributes to the practice of safety management. By analyzing the relationship among SL, SC, and SP, as well as the role of two moderating variables (economic level and industry risk degree) in the SL–SP relationship, a way to improve SP is revealed. The results suggest that enterprise safety leaders can choose to prefer transformational leadership to improve employee SP and improve the internal SC in combination with the industry risk degree and the economic level of the country where the enterprise is located.

## Conclusion

This study revealed the inner relationship between SL and SP and identified indirect mechanisms, such as the mediating effect of SC and the moderating effect of economic level and industry risk degree. A total of 35 records related to SL and SP were extracted from 33 papers published in the last 21 years. A model of how different SL styles related to SP was proposed and tested *via* a meta-analysis of CMA software.

The analysis results showed that SL has a positive impact on SC and employees’ SP and the latter’s impact is stronger. Compared with safety transactional leadership, STFL has a more significant impact on employees’ SP. However, the relationship between SPL and SP was not supported. SC plays a partial mediating role between transformational SL and employee SP. Again, the impact of SL on SP is affected by the economic level and the risk degree of the operating industry. Under the condition of a developed economic level or high-risk industry, SL has a greater influence on employees’ SP.

These findings may contribute to the future development of safety management theory and practice, especially in optimizing SL, improving employee SP, and promoting the improvement of safety performance.

## Data Availability Statement

The raw data supporting the conclusions of this article will be made available by the authors, without undue reservation.

## Author Contributions

LZ: conceptualization and methodology, original draft preparation, writing—reviewing and editing, and data acquisition and analysis. DY: supervision and sponsor. SL: supervision, sponsor, and editing. EN: data acquisition and editing. All authors contributed to the article and approved the submitted version.

## Conflict of Interest

The authors declare that the research was conducted in the absence of any commercial or financial relationships that could be construed as a potential conflict of interest.

## Publisher’s Note

All claims expressed in this article are solely those of the authors and do not necessarily represent those of their affiliated organizations, or those of the publisher, the editors and the reviewers. Any product that may be evaluated in this article, or claim that may be made by its manufacturer, is not guaranteed or endorsed by the publisher.
