# Supplementary material for: The Effect of Safety Leadership on Safety Participation of Employee: A Meta-Analysis
Source: Front Psychol. 2022 Jun 16;13:827694. doi: 10.3389/fpsyg.2022.827694 (PMC9246271; doi:10.3389/fpsyg.2022.827694)
Supplement: Supplementary file 1 [file Table_1.docx]

# Appendix: Studies included in this meta-analysis

| **main reseacher** | **publish date** | **country/region** | **Industrial sector** | **variables involved** | **sample number** | **correlation coefficients** |
| --- | --- | --- | --- | --- | --- | --- |
| Mark A. Griffin | 2000 | Australia | manufacturing and mining | safety leadership,safety participation | 1453 | 0.605 |
| Dov Zohar | 2002 | Israel | metal processing | safety leadership,safety climate | 411 | 0.263 |
| Julian Barling | 2002 | Canada | restaurant | safety leadership,safety climate | 174 | 0.42 |
| Bernard M. Bass | 2003 | US | army | safety leadership,safety climate | 72 | 0.185 |
| David A. Hofmann | 2003 | US | army | safety leadership,safety climate,safety participation | 94 | 0.516 |
| Sharon Clarke | 2006 | UK | glassware manufacturing | safety leadership,safety climate,safety participation | 105 | 0.277 |
| E. Kevin Kelloway | 2006 | Canada | - | safety leadership,safety climate | 158 | -0.003 |
| Anders Skogstad | 2007 | Norway | - | safety leadership,safety climate | 2273 | -0.35 |
| Shang Hwa Hsu | 2008 | Taiwan | Taiwan oil refinery | safety leadership,safety participation | 295 | 0.465 |
|  |  |  |  |  | 256 | 0.403 |
| Shama Didla | 2009 | UK | oil and gas production | safety climate,safety participation | 27 | 0.519 |
| SM Conchie | 2009 | UK | construction industry | safety leadership,safety participation | 139 | 0.38 |
| Chin-Shan Lu | 2010 | China | container terminal | safety leadership,safety participation | 336 | 0.5 |
| Dov Zohar | 2010 | Israel | metal food plastics and chemical industries | safety leadership,safety climate | 3952 | 0.22 |
| Michelle Inness | 2010 | US | - | safety leadership,safety participation | 159 | 0.51 |
| Nielsen K | 2011 | Denmark | service industry | safety leadership,safety participation | 58 | 0.51 |
| Jane Mullen | 2011 | Canada | business and psychology | safety leadership,safety participation | 241 | 0.309 |
|  |  |  | health care |  | 491 | 0.129 |
| Tsung-Chih Wu | 2011 | Taiwan | petrochemical | safety leadership,safety climate | 521 | 0.52 |
| DU Xuesheng | 2012 | China | coalmines | safety leadership,safety climate,safety participation | 450 | 0.448 |
| E.A. Kapp | 2012 | US | road building and manufacturing | safety leadership,safety climate | 555 | 0.25 |
| Mark A. Griffin | 2013 | Australia | - | safety leadership,safety participation | 254 | 0.104 |
| Beatriz Fernández-Muñiz | 2014 | Spain | construction industry and sevices | safety leadership,safety climate,safety participation | 188 | 0.485 |
| Fan Zhou | 2015 | China | power supplying | safety climate,safety participation | 178 | 0.25 |
| Todd D. Smith | 2016 | US | fire service | safety leadership,safety climate,safety participation | 398 | 0.129 |
| Lixin Jiang | 2016 | US | - | safety leadership,safety climate,safety participation | 171 | 0.017 |
| Yuzhong Shen | 2017 | Hong Kong | construction | safety leadership,safety climate,safety participation | 292 | 0.35 |
| Jane Mullen | 2017 | Canada | trade | safety leadership,safety participation | 140 | 0.37 |
| Kriengsak Panuwatwanich | 2017 | Australia | construction industry | safety climate,safety participation | 295 | 0.84 |
| Chunlin Wu | 2017 | China | construction | safety leadership,safety participation | 235 | 0.725 |
| Shezeen Oah | 2018 | korea | manufacturing | safety leadership,safety climate | 376 | 0.66 |
| Xiaohua Bian | 2019 | China | construction industry | safety leadership,safety climate,safety participation | 260 | -0.174 |
| Yujingyang Xue | 2020 | China | petrochemical industry | safety leadership,safety climate,safety participation | 155 | 0.653 |
| Lianhua Cheng | 2020 | China | coalmines | safety leadership,safety participation | 300 | 0.28 |
| AK Schopf | 2021 | Belgium | air traffic | safety leadership,safety participation | 287 | 0.14 |
